# Physiological and Comparative Genomic Analysis of *Arthrobacter* sp. SRS-W-1-2016 Provides Insights on Niche Adaptation for Survival in Uraniferous Soils

**DOI:** 10.3390/genes9010031

**Published:** 2018-01-11

**Authors:** Ashvini Chauhan, Ashish Pathak, Rajneesh Jaswal, Bobby Edwards, Demario Chappell, Christopher Ball, Reyna Garcia-Sillas, Paul Stothard, John Seaman

**Affiliations:** 1 Environmental Biotechnology and Genomics Laboratory, School of the Environment, 1515 S. Martin Luther King Jr. Blvd., Suite 305B, FSH Science Research Center, Florida A&M University, Tallahassee, FL 32307, USA; ashish1.pathak@famu.edu (A.P.); rajneesh.jaswal@famu.edu (R.J.); bobby1.edwards@famu.edu (B.E.); 2 Department of Biology, College of Science and Technology, 1610 S. Martin Luther King Blvd., Florida A&M University, Tallahassee, FL 32307, USA; demariochappell@yahoo.com; 3 Department of Biological Sciences, Life Science Building, Alabama State University, 915 S Jackson Street, Montgomery, AL 36101, USA; Cball4325@myasu.alasu.edu; 4 School of Life Sciences, College of Liberal Arts and Sciences, Arizona State University, 723 E 6th St, Tempe, AZ 85281, USA; rgarci35@asu.edu; 5 Department of Agricultural, Food and Nutritional Science, University of Alberta, Edmonton, AB T6G 2P5, Canada; stothard@ualberta.ca; 6 Savannah River Ecology Laboratory, University of Georgia, Aiken, SC 29802, USA; seaman@srel.uga.edu

**Keywords:** bioremediation, uranium, nickel, whole genome sequencing, resistome, comparative genomics, *Arthrobacter*

## Abstract

*Arthrobacter* sp. strain SRS-W-1-2016 was isolated on high concentrations of uranium (U) from the Savannah River Site (SRS) that remains co-contaminated by radionuclides, heavy metals, and organics. SRS is located on the northeast bank of the Savannah River (South Carolina, USA), which is a U.S. Department of Energy (DOE) managed ecosystem left historically contaminated from decades of nuclear weapons production activities. Predominant contaminants within the impacted SRS environment include U and Nickel (Ni), both of which can be transformed microbially into less toxic forms via metal complexation mechanisms. Strain SRS-W-1-2016 was isolated from the uraniferous SRS soils on high concentrations of U (4200 μM) and Ni (8500 μM), but rapid growth was observed at much lower concentrations of 500 μM U and 1000 μM Ni, respectively. Microcosm studies established with strain SRS-W-1-2016 revealed a rapid decline in the concentration of spiked U such that it was almost undetectable in the supernatant by 72 h of incubation. Conversely, Ni concentrations remained unchanged, suggesting that the strain removed U but not Ni under the tested conditions. To obtain a deeper understanding of the metabolic potential, a draft genome sequence of strain SRS-W-1-2016 was obtained at a coverage of 90×, assembling into 93 contigs with an N50 contig length of 92,788 bases. The genomic size of strain SRS-W-1-2016 was found to be 4,564,701 bases with a total number of 4327 putative genes. An in-depth, genome-wide comparison between strain SRS-W-1-2016 and its four closest taxonomic relatives revealed 1159 distinct genes, representing 26.7% of its total genome; many associating with metal resistance proteins (e.g., for cadmium, cobalt, and zinc), transporter proteins, stress proteins, cytochromes, and drug resistance functions. Additionally, several gene homologues coding for resistance to metals were identified in the strain, such as outer membrane efflux pump proteins, peptide/nickel transport substrate and ATP-binding proteins, a high-affinity nickel-transport protein, and the *spoT* gene, which was recently implicated in bacterial resistance towards U. Detailed genome mining analysis of strain SRS-W-1-2016 also revealed the presence of a plethora of secondary metabolite biosynthetic gene clusters likely facilitating resistance to antibiotics, biocides, and metals. Additionally, several gene homologous for the well-known oxygenase enzyme system were also identified, potentially functioning to generate energy via the breakdown of organic compounds and thus enabling the successful colonization and natural attenuation of contaminants by *Arthrobacter* sp. SRS-W-1-2016 at the SRS site.

## 1. Introduction

Savannah River Site (SRS) located in Aiken, South Carolina, functioned as a nuclear materials production facility for the U.S. Department of Energy (DOE), where metal-clad uranium (U) targets were used in the production of plutonium [1]. From 1954 to 1982, SRS wastewater originating from the metal plating and fabrication processes was released directly into Tims Branch, a second-order stream. Because of these activities, large quantities of both U (depleted as well as naturally-occurring) and Nickel (Ni) were released and deposited into stream sediments and an abandoned farm pond- the Steeds Pond, that served as a natural settling basin along Tims Branch [1,2]. Although contaminant distribution is heterogeneous in the Steeds Pond sediments, U and Ni concentrations are found upwards of 1000 mg/kg [2]. To gauge in-situ remediation (natural attenuation) and recommend appropriate strategies for the decontamination of SRS contaminated ecosystems, it is necessary to obtain a deeper understanding of the mechanisms exhibited by the native soil microbiota that have evolved to resist metals and radionuclides. In fact, microorganisms exposed to the contaminant stresses not only have the unparalleled ability to survive in radionuclide contaminated environments, but also potentially reduce the toxicity of both U [3,4,5] and Ni [6], respectively. Microbially-mediated mechanisms for the reduction of U toxicity include bioreduction, biosorption, biomineralization, and bioaccumulation [4]. For Ni, most commonly known resistance mechanisms are mediated by efflux pumps such as cnr CBA (cobalt-nickel resistance) from *Cupriavidus metallidurans* CH34, NccCBA (Nickel-cobalt-cadmium) and NreB (nickel resistance) from *Achromobacter xylosoxidans* 31A, and CznABC (Cadmium-zinc-nickel) from *Helicobacter pylori* [6]. However, the genomic mechanisms underpinning U and Ni conversions into the less mobile and less toxic forms continue to remain unclear.

Soil samples for this study were collected from site 101 located within the Tims Branch system. At this site, U concentrations are typically present between 700 and 800 ppm [5], which are considered high U concentrations invoking the criteria of Mumtaz et al. [7]. Therefore, this site represents an opportunity to study genome-enabled mechanisms recruited by the SRS-native microorganisms, facilitating their survival in co-contaminated environments. In fact, the stress posed by environmental contaminants facilitates the recruitment of genes by horizontal gene transfer mechanisms that enable the microbial cells to not only resist, but also bioremediate, the contaminants, mostly by the synthesis of proteins for cellular survival [8]. Some examples of such genomic-mechanisms include efflux systems, the presence of metal resistant genes, detoxification genes, and biosorption/bioaccumulation of the contaminant at or within the bacterial cell membrane [9].

Furthermore, a growing body of research is demonstrating that metal contaminants in the environment have the propensity to co-select for antibiotic resistance within the naturally-occurring microbiota [10]. Co-selection occurs through the following mechanisms: (a) co-resistance- when there is physical proximity of the resistome encoding antibiotics and/or metal resistance, e.g., on the same genetic element (plasmid) or in the same cell (e.g., *merA* and *KPC* beta-lactamase); (b) cross-resistance- when a single resistome functions to provide efflux and antibiotic resistance (e.g., *mdrL* confers resistance to metals, such as zinc, cobalt, and chromium, along with antibiotics, such as erythromycin, josamycin, and clindamycin) [11]; and (c) co-regulatory resistance- in this situation, multiple resistance genes conferring resistance to different toxic compounds, including antibiotics, biocides, and metals, are controlled by a single regulatory gene element (e.g., *czcR* regulating the expression of the *CzcCBA* efflux pump, resulting in the resistance to zinc, cadmium, and cobalt, along with co-regulating resistance to antibiotic carbapenems) [12].

To further understand environmentally-relevant genomic mechanisms that underpin microbial survival in radionuclide and heavy metal-rich ecosystems, we recently isolated several bacterial strains in the presence of high concentrations of both U and Ni [13]. 16S-gene based analysis revealed that the isolated strains mainly belonged to *Burkholderia* spp. and *Arthrobacter* spp.—genera demonstrated to serve as bioindicators of environmental contamination, as well as agents of bioremediation, especially U [14]. In fact, *Arthrobacter* spp., were found to be predominant in the U-contaminated Hanford site [15], which is another DOE site in Washington State having a similar U contamination history as the SRS site. *Arthrobacter* spp., have also been isolated from ecosystems under extreme environmental stresses, such as a nuclear waste plume amd U mined site, as well as heavy metal contaminated habitats [14,16,17,18,19,20]. Therefore, it appears that *Arthrobacter* spp., have recruited strong genomic traits to be able to colonize and survive in radionuclide and metal contaminated habitats. Understanding the basis of metal-microbial interactions, therefore, is a prerequisite for the successful management and/or rehabilitation of nuclear-legacy contaminated environments.

We recently characterized the genomic traits of *Burkholderia* sp. strain SRS-W-2-2016, a bacterial strain isolated from the same cohort as *Arthrobacter* sp. SRS-W-1-2016 [13]; which revealed the presence of several gene homologues previously demonstrated to code for the resistance of heavy metals and radionuclides, forming the basis of this study. Specifically, in this study, we report several ecologically relevant genomic traits present in *Arthrobacter* sp. SRS-W-1-2016, including a suite of substrate binding proteins, permeases, transport regulators, and efflux pumps- likely working in concert to potentially detoxify toxic metals and thus facilitating the natural attenuation of contaminants within the SRS impacted ecosystem. Such genome-enabled studies will facilitate a deeper understanding of heavy metal and antibiotic resistance and hydrocarbon degradative mechanisms in complex, mixed contaminant habitats and provide recommendations for the environmental stewardship of anthropogenically-impacted environments.

## 2. Results and Discussion

### 2.1. Uranium and Nickel Depletion Potential of Arthrobacter sp. SRS-1-W-2016 

**Figure 1:**
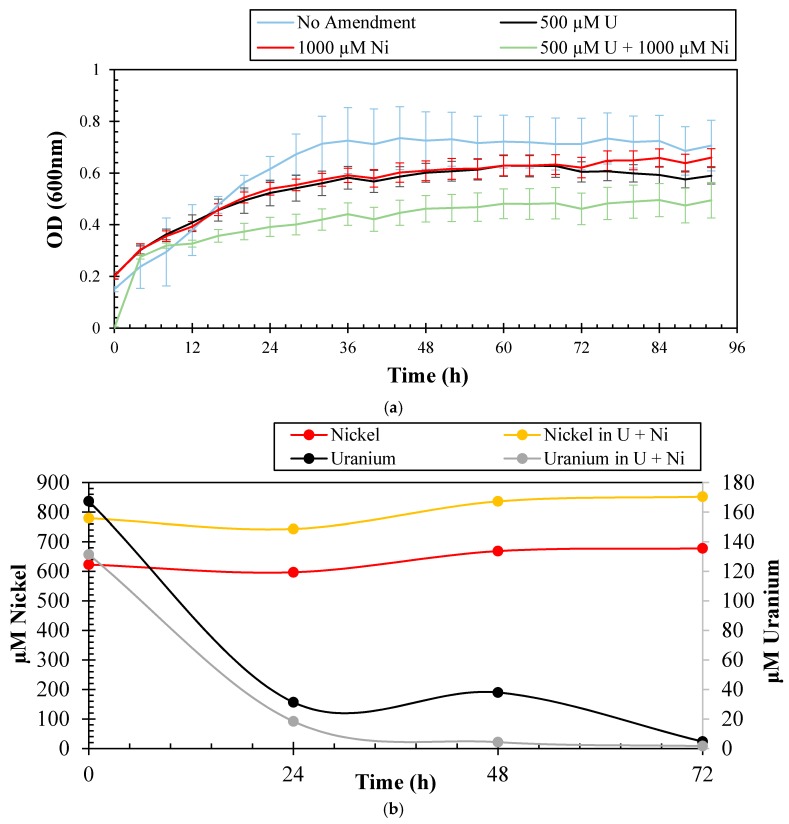

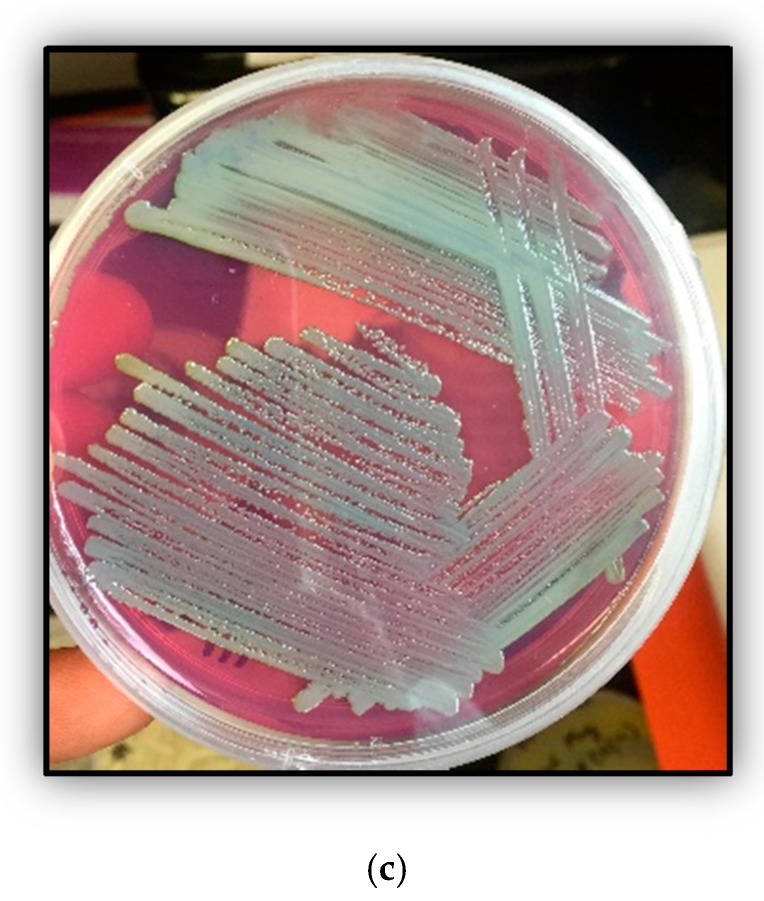
Shown are (**a**) growth experiments to determine the resistance abilities of *Arthrobacter* sp. strain SRS-W-1-2016 to Uranium (U) and Nickel (Ni), respectively. Growth of strain is shown in 4M media without amendments, as well as supplemented with 500 µM U, 1000 µM Ni and, and in the combination of 500 µM U and 1000 µM Ni, respectively; (**b**) shown are the measured concentrations of U and Ni over a three-day period that were spiked in microcosms containing strain SRS-W-1-2016; (**c**) histochemical plate-based growth assay to show phosphatase enzyme-based U-precipitation potential of strain SRS-W-1-2016. Bacteria that display a phosphatase-positive phenotype appear bright green on Tryptose Phosphate Methyl GreenTPMG agar plate.

The ability of bacterial strains to resist U toxicity has been previously demonstrated. In fact, 90% of bacteria representative of taxa Firmicutes, Gammaproteobacteria, Actinobacteria, Bacteroidetes, and Betaproteobacteria identified from the Uranium mining site of Domiastat, India, were found to be resistant to a high U concentration of 4 mM [21]. Kulkarni et al. [22] isolated two phosphatase producing bacteria- *Escherichia coli* and *Deinococcus radiodurans*, which were found to grow in as high as 20 mM U. *Burkholderia fungorum* isolated from Rifle, Colorado, was found to grow on 10 µM U [23]. Similarly, several strains resistant to particularly high concentrations of Ni have been isolated from various contaminated sources. Alboghobeish et al. [24] isolated several strains, namely *Methylobacterium* sp. ATHA7 and *Klebsiella oxytoca* ATHA6 resistant to 24 mM and 16 mM Ni, respectively, from industrial sewage. *Cupriavidus taiwanensis* isolated from the root nodules of *Mimosa pudica* was found to be resistant to 1.5 mM Ni, along with some other heavy metals [25]. To determine the resistance potential of strain SRS-1-W-2016 against U and Ni, different concentrations of these metals were supplemented in 4M media and the growth results are presented in Figure 1a. Strain SRS-1-W-2016 could resist up to 4.2 mM of U and 8.5 mM of Ni, which are the typical concentrations of these metals in the sampling location [13], albeit, rapid growth was only observed at much lower concentrations of 500 µM U and 1000 µM Ni, respectively; a stationary phase was reached in 48 h. A similar trend was shown in the presence of 1000 µM Ni. When grown in the combination of 1000 µM Ni and 500 µM U, the stationary phase was only achieved after 60 h of incubation, potentially due to the combined stress of both U and Ni. In the native soil environment from where the strain was isolated, both U and Ni are the major co-contaminants and therefore the growth profile obtained for these co-contaminants provides a more accurate representation of the strain’s metal resistance ability.

To further evaluate the bioremediation potential of strain SRS-1-W-2016 for U and Ni separately, as well as in combination, microcosms were established for quantification of the amended metals by inductively coupled plasma-mass spectrometry (ICP-MS) and these results are shown in Figure 1b. It is clear that the initial concentration of U in the microcosms declined rapidly in merely 24 h and most of the spiked U declined completely in the supernatant by 72 h, strongly suggesting that strain SRS-1-W-2016 has the ability to quickly biotransform and/or bioremediate U. Several mechanisms are recognized by which microbes are able to transform and resist toxicity posed by extracellular U. Some of these mechanisms include reductive precipitation by outer membrane cytochromes, conductive pili, or spores, surface adsorption by exopolysaccharide (EPS) or S-layers, or precipitation with phosphate compounds, respectively. To evaluate the mechanism utilized by strain SRS-1-W-2016 to grow in the presence of U, we used the histochemical growth assay on tryptose phosphate methyl green (TPMG) media. We observed an intense green coloration formed by strain SRS-W-1-2016 during growth on an Luria Bertani (LB) plate supplemented with U (Figure 1c), indicating the activity of phosphatase enzyme-based bioremediation of U. Surprisingly, no decline in the concentrations of the amended Ni was observed under the tested conditions (Figure 1b). It is highly likely that efflux mechanisms were employed by the strain to prevent the cellular uptake of Ni and hence no bioremediation activity against Ni was documented.

### 2.2. Genome-Centric Evaluation of Strain SRS-W-1-2016 

**Figure 2:**
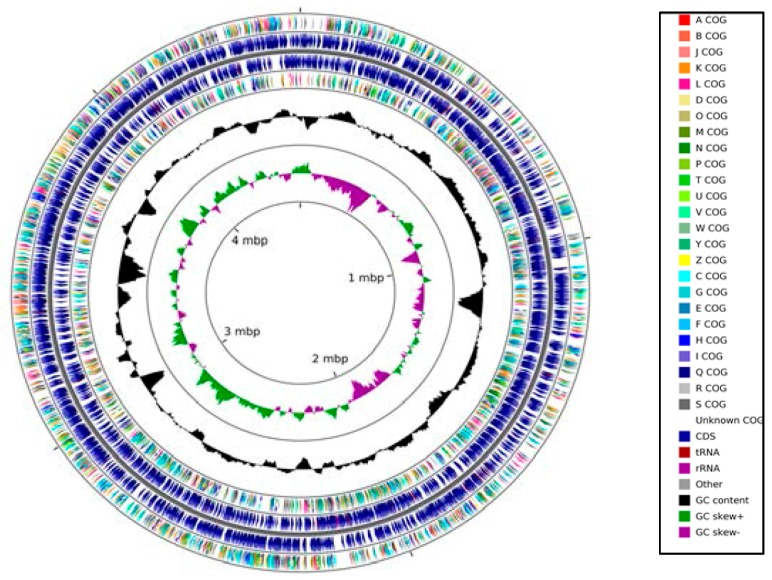
Circular genome map of *Arthrobacter* sp. strain SRS-W-1-2016 with the first (outermost) and fourth rings depicting clusters of orthologous groups of proteins (COG) categories of protein coding genes on the forward and reverse strands, respectively. The second and third rings show the locations of protein coding, tRNA, and rRNA genes on the forward and reverse strands, respectively. The black plot depicts GC content with the peaks extending towards the outside of the circle representing GC content above the genome average, whereas those extending towards the center mark segments with GC content lower than the genome average. The innermost plot depicts GC skew. Both base composition plots were generated using a sliding window of 50,000 nt.

The genome size of strain SRS-W-1-2016 was predicted to be approximately 4,564,701 bases with a G+C content of 64.1. Moreover, the strain contained a total of 4327 coding sequences, with 58 total RNA genes, 57 tRNA genes, and two copies of the 16S rRNA genes, respectively. A circular genomic map of strain SRS-W-1-2016 using the 93 contigs with an N50 contig length of 92,788 bases is shown in Figure 2.

**Figure 3:**
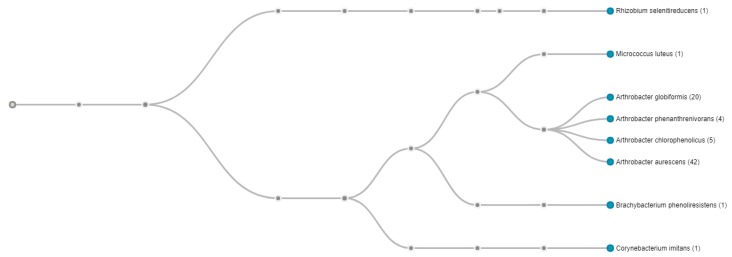
Whole genome sequence-based phylotree of *Arthrobacter* sp. strain SRS-W-2-2016 relative to a cohort of 24 *Arthrobacter* spp., for which genome sequences are available. The highest taxonomic affiliation of strain was found with *Arthrobacter aurescens* TC1 based on the highest number of whole genome sequence reads (42; shown in parenthesis) that clustered with this group. The tree was generated based on the presence of the Pfam function category amongst *Arthrobacter* spp. available in the integrated microbial genome expert review (IMG/er) database. Displayed nodes are those with at least one read (<1% of classified reads).

Further genome-centric evaluation of strain SRS-W-1-2016 revealed that out of 4318 total protein coding genes, 64.75% genes associated with clusters of orthologous groups of proteins (COGs); 74.34% genes annotated to protein coding function predictions; 24.36% genes annotated as protein coding genes with enzyme production; and 25.59% genes were connected to Kyoto Encyclopedia of Genes and Genomes (KEGG) pathways. Note that COGs represent proteins that are an ortholog or direct evolutionary counterpart among bacterial genomes as they evolve over time. When the whole genome sequence of strain SRS-W-1-2016 was taxonomically analyzed using the One Codex RefSeq database, 52.5% of classified reads (readcount 42) clustered with *Arthrobacter aurescens* TC1, followed by 25% (readcount 20) with *Arthrobacter globiformis* NBRC 12,137, respectively (Figure 3). Two-way average nucleotide identity (ANI) between strain SRS-W-1-2016 and the closest relative *Arthrobacter aurescens* TC1 was estimated to be 80.28%, (SD: 5.24%; Supplementary Figure S1), from 3101 fragments that were included in this analysis, indicating that SRS-W-1-2016 may be a new species that is distinctly different than *Arthrobacter aurescens* TC1. Similar to the ANI, when digital DNA-DNA hybridization (dDDH) was estimated using the Genome-to-Genome Distance web service, it was estimated that strain SRS-W-1-2016 was 21.20% similar to *Arthrobacter aurescens* TC1 (data not shown). Note that according to the pipeline used, DDH > 70% (via logistic regression) would mean that the two genomes belong to the same species. Together, this is strong evidence that SRS-W-1-2016 has a unique taxonomic position relative to other members of *Arthrobacter* species for which whole genome sequence data is available.

*Arthrobacter* species, which are members of the soil actinobacteria, are ubiquitously distributed in diverse ecological habitats, and are known for their ability to survive in extreme conditions such as UV irradiation and radioactivity [26]. Efficient molecular adaptation mechanisms in *Arthrobacter* enhance the stability of nucleic acids, proteins, and lipids, allowing bacteria to survive in extreme conditions [27], such as the SRS co-contaminated soils. Some species of *Arthrobacter* such as *Arthrobacter crystallopoietes* [28] and *Arthrobacter chlorophenolicus* [29] have been utilized for the bioremediation of chromium and 4-chlorophenol in contaminated soils. Generally, *Arthrobacter* is known to grow on complex aromatic substrates such as hydroxybenzoates, pyridine, and picoline [30], which is a unique biodegradative ability of this group of soil bacteria. In fact, as stated earlier in this study, *Arthrobacter* spp., have been shown to successfully colonize habitats characterized by extreme environmental stresses, including nuclear waste plume heavy metal contaminated sites, as well as uraniferous niches, such as the U-impacted DOE Hanford site [15], which points to the genomic-mechanisms possessed by *Arthrobacter* spp., for the colonization of metal-rich ecosystems.

To further analyze the genome of strain SRS-1-W-2016, a KEGG -based hierarchical functional gene clustering analysis was performed (Table 1), which revealed the presence of a total of 1124 KEGG-associating genes; the top five categories belonged to carbohydrate metabolism (284 genes; 25.27%); amino acid metabolism (256 genes; 22.78%); energy metabolism (135 genes; 12.01%); vitamin and cofactor metabolism (128 genes; 11.39%); and membrane transport (105 genes; 9.34%), respectively. Additionally, a total of 2844 protein coding genes associated with COGs in strain SRS-W-1-2016 were found with further classification into 25 categories, with the most abundant COG subsystems as follows: carbohydrate transport and metabolism; and amino acid transport and metabolism. The transcription of many of these features suggests the propensity of this strain to engage in high metabolic activity in its native contaminated environment. These analyses provide a strong foundation for the presence of several genome-enabled metabolic and catabolic processes in *Arthrobacter* sp. strain SRS-W-1-2016 which likely play a significant role in its successful colonization and survival within the SRS co-contaminated soil habitat.

### 2.3. Genes Potentially Conferring Metal Resistance in Strain SRS-W-1-2016 

One notable attribute of the *Arthrobacter* genus is their ability to degrade xenobiotic compounds and resistance to heavy metals [26,31,32,33,34], including U and Ni [27]. Genome-centric assessment of strain SRS-W-1-2016 revealed the presence of several gene homologues previously demonstrated to play a part in the resistance against heavy metal/radionuclides, such as several efflux and MFS transporters, P-type ATPase translocators, and heavy metal-responsive transcriptional regulators.

**Figure 4:**
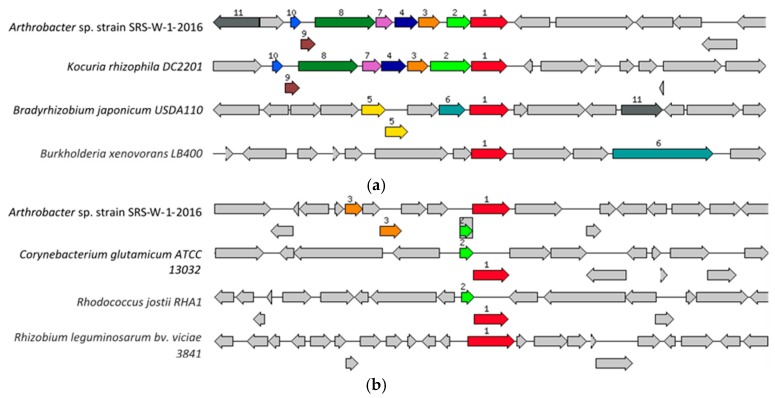
Shown are (**a**) the chromosomal region of the cobalt/zinc/cadmium subsystem gene (1056 bp) in *Arthrobacter* sp. strain SRS-W-2-2016 relative to four other organisms and (**b**) the *HoxN*/*HupN*/*NixA* family nickel/cobalt transporter gene (1089 bp) in *Arthrobacter* sp. strain SRS-W-2-2016 relative to four other organisms. The graphic is centered on the focus gene, which is red and numbered 1. Sets of genes with a similar sequence are grouped with the same number and color. Genes whose relative position is conserved in at least four other species are functionally coupled and share gray background boxes.

Specifically, for metals resistance, the genome of strain SRS-W-1-2016 contains the high affinity transport system for nickel/cobalt (*NiCoT* gene; *HoxN*/*HupN*/*NixA* family), as well as the cobalt/zinc/cadmium resistant protein (*CzcD*). The *NiCoT* system, shown relative to four homologous gene systems (Figure 4a), was found to occur as a 1056 bp fragment (contig41300; fig|6666666.232708.peg.2084), while the *CzcD* subsystem, (Figure 4b) was a 1089 bp gene fragment (contig51456; fig|6666666.232708.peg.2658), respectively. In addition to these genes, bacterial resistance to metals is also governed by genes for the resistance, nodulation, and cell division (RND) family proteins- all part of transenvelope protein complexes which detoxify the cellular environment by exporting toxic metal cations from the periplasm to the outside. Several RND-type efflux gene homologues were found interspersed within the genome of strain SRS-W-1-2016 (data not shown). Additionally, the ABC-type transporters and heavy-metal transport/detoxification proteins were abundantly present within the genome of strain SRS-W-1-2016, potentially maintaining metal homeostasis and survival in the SRS co-contaminated soil habitat.

There are several mechanisms by which bacteria can reduce the mobility and hence toxicity of U and one process that shows bioremediation potential is by a phosphatase enzyme mediated biotransformation of U (VI) into phosphate-minerals [27,29]. It has been demonstrated that microbially-mediated alkaline or acid phosphatase activities can result in the precipitation of more than 90% of soluble U, resulting in the formation of a wide array of uranyl phosphate minerals [30]. Therefore, the microbially-mediated formation of uranyl phosphate from U (VI) warrants further research for the control of U mobility in U.S. DOE contaminated environments. To identify the presence of the phosphatase enzyme within the genome of strain SRS-W-1-2016, we manually queried the annotated genome, revealing the presence of two acid phosphatases (NCBI gene accession # OOP63314 and OOP61946), as well as an alkaline phosphatase (NCBI gene accession # OOP64358). Moreover, we also identified the *spoT* gene (contig50361; fig|6666666.232708.peg.2556) in strain SRS-W-1-2016 (data not shown); the *spoT* gene encodes a guanosine tetraphosphate (ppGpp) hydrolase/synthetase which was recently implicated in the resistance of U by *Caulobacter crescentus,* but only under carbon starvation conditions [35]. Collectively, these mechanisms likely render the metabolic ability to strain SRS-W-1-2016 to not only survive in the SRS soils, but also perform critical biodegradative functions.

Moreover, in addition to the metal-related genes, several genes that potentially confer the ability to degrade organic contaminants were also identified in strain SRS-W-1-2016; especially oxygenase enzymes (data not shown). This finding is relevant because oxygenases have been previously shown to co-metabolize the degradation of trichloroethylene (TCE), one of the widespread organic contaminants identified in SRS-impacted habitats [36]. Therefore, it is likely that strain SRS-W-1-2016 is metabolically active in the biodegradation and biomineralization of not only metals, but also hydrocarbons in the SRS soils.

### 2.4. Resistome of Arthrobacter sp. Strain SRS-W-1-2016 

When the genome of strain SRS-W-1-2016 was mined for the presence of antibiotic resistance genes (the resistome), gene determinants for the following drug resistance were noted (percent identity with matching region are shown in the parenthesis): fluoroquinolone (36%), isoniazid (46%), fosfomycin (43%), aminocoumarin (66%), mupirocin (54%), and rifamycin (33%), respectively. Several other genes that potentially confer antibiotic resistance were also identified, including the antibiotic target protection protein (36%), gene involved in self-resistance to antibiotic (66%), efflux pump complex or subunit conferring antibiotic resistances [(39% with *ImrB* and 40% with *ImrD*—both confer resistance to lincosamides) and 55% with *novA* (resistance to novobiocin)], antibiotic resistant gene variants or mutants, and the antibiotic inactivation enzyme (Supplementary Figure S2). Because a lower degree of homology, ranging between 36% to 66%, was observed between the resistome of strain SRS-W-1-2016 with the previously characterized antibiotic resistance genes, there is a strong possibility of a novel resistome that has developed in this strain, likely due to the selective pressure of heavy metals, U, and organic compounds.

Moreover, when the genome of strain SRS-W-1-2016 was evaluated for the presence of biosynthetic gene clusters (BCs) responsible for the synthesis of secondary metabolites (SMs), seven BCs with 150 associated genes were identified (Supplementary Figure S3). The predominant BC identified from strain SRS-W-1-2016 affiliated with the well-known nonribosomal peptide synthetase (NRPS)- type III polyketide synthase (T3PKS), with a total of 69 pfams; other BCs were also identified for bacteriocin (27 pfams) and butyrolactone (11 pfams). Interestingly, many pfams that were found to be overrepresented across the seven BCs identified in strain SRS-W-1-2016 have been shown to play a role in metals-microbe interactions (appear as red boxes in the heat map; Supplementary Figure S3).

Notably, a total of 46 secondary metabolite clusters were identified using the antiSMASH pipeline with genes homologous to the biosynthesis of (similarity shown in parenthesis): Amphotericin (23%); Streptomycin (15%); Arsenopolyketides (12%); Echosides (11%); Guadinomine (11%); Thiolutin (8%); Kanamycin (7%); Chlorizidine (7%); Pristinamycin (5%); Meilingmycin (2%); Lobosamide (4%); Enduracidin (4%); and Herboxidiene (2%), respectively. Overall, this resistome analysis depicts the broad metabolic and biosynthetic potential of *Arthrobacter* sp. strain SRS-W-1-2016.

### 2.5. Prediction of Phenotypic Traits in Arthrobacter sp. Strain SRS-W-1-2016 

**Figure 5:**
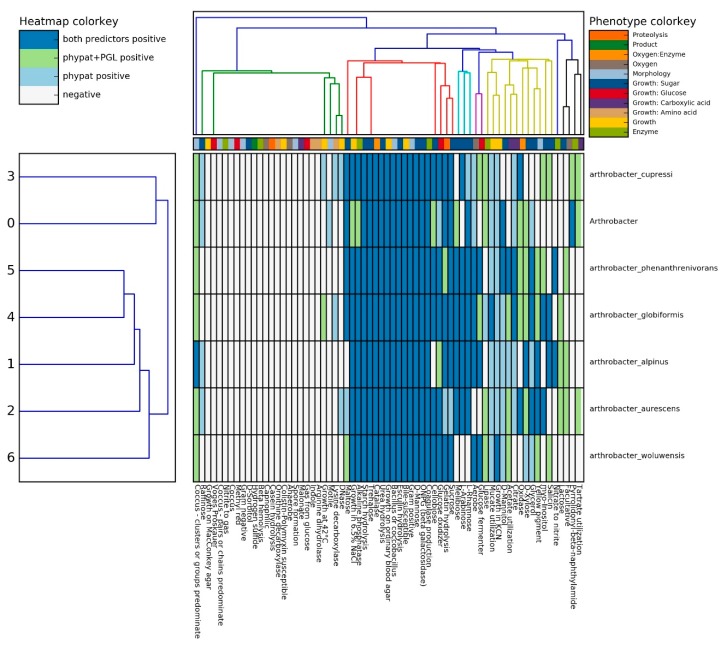
In-silico phenotypic comparison of *Arthrobacter* sp. SRS W-1-2016 with six closely related *Arthrobacter* spp. using the Traitar pipeline.

To further probe the genotype to phenotype traits, a recently developed pipeline was utilized for strain SRS-W-1-2016 relative to several other *Arthrobacter* spp., which revealed the ability for sugar hydrolysis such as maltose, trehalose, mannitol, rhamnose, and mannose by both the classifiers, while glucose, xylose, and cellobiose were confirmed by only one of the classifiers during Traitar analysis of *Arthrobacter* sp. strain SRS-W-1-2016 (Figure 5). However, certain traits such as the utilization of arabinose, fermentation of glucose, and utilization of acetate, which were observed in other *Arthrobacter* strains, were absent in strain SRS-W-1-2016.

Using the phypate + PGL predictor built in the pipeline also confirmed the presence of the alkaline phosphatase enzyme in *Arthrobacter* sp. strain SRS-W-1-2016, which may be associated with U precipitation and could be one of the factors to confer resistance to the bacterium at a toxic U concentration. Interestingly, *Arthrobacter* sp. strain SRS-W-1-2016 was the only motile bacterium relative to the other selected *Arthrobacter* spp. group of bacteria, according to the phypat predictor; motility is a useful trait that can facilitate bacterial chemotaxis and enhance biodegradative functions [37].

### 2.6. Genomic Islands in Arthrobacter sp. Strain SRS-W-1-2016 

Another interesting genomic trait of strain SRS-W-1-2016 is the presence of several genomic islands (GEIs). Bacterial genomes consist of a set of core genes encoding for essential metabolic functions which are supplemented with genes acquired via horizontal gene transfer (HGT) mechanisms that may render additional metabolic functions to the recruiting bacteria such as adaptive traits and genomic plasticity, facilitating evolutionary survival [38]. GEIs have been more commonly known to render virulence or antibiotic resistance to the host bacteria, but more recently, whole genome sequencing studies have also revealed other GEI-encoded functional traits classified into the following four broad categories - pathogenicity islands (PAIs), that code for virulence genes; metabolic islands (MIs), genes for the biosynthesis of secondary metabolites; resistance islands (RIs), genes that code for resistance- typically against antibiotics; and symbiotic islands (SIs), facilitating symbiotic associations of the host with other micro- and macroorganisms.

**Figure 6:**
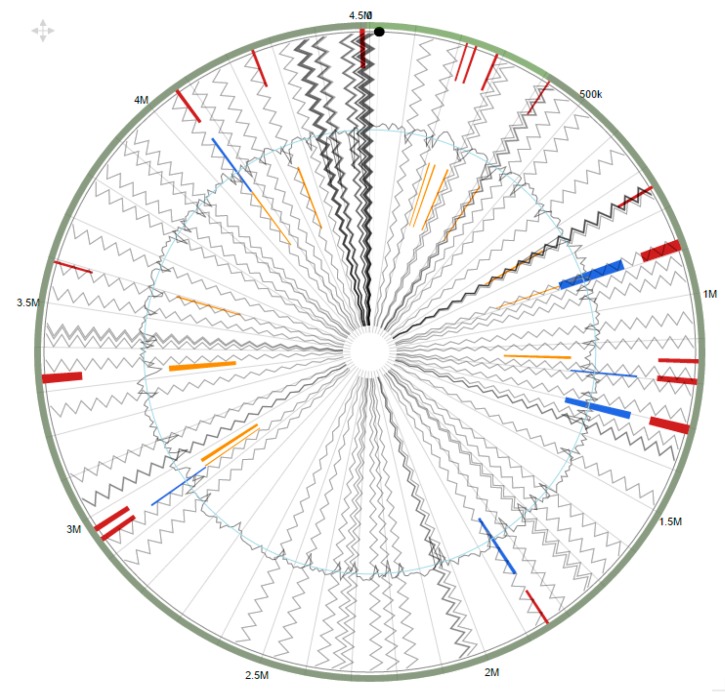
Shown are the putative genomic islands (GEIs) of *Arthrobacter* sp. strain SRS-W-1-2016. The outer green circle represents the scale line in Mbps and GEIs obtained from each of the following methods are shown in color: SIGI-HMM (orange), IslandPath-DIMOB (blue), and integrated detection (red), respectively.

Interestingly, several genomic islands were identified in strain SRS-W-1-2016 when compared against the complete genome of *Arthrobacter aurescens* TC1 as the reference strain (Figure 6). When the identified GEI homologues in strain SRS-W-1-2016 were further analyzed by BLAST, several of them closely affiliated with genes previously implicated in the resistance of metals, as well as the biodegradation of contaminant hydrocarbons, suggesting the strong possibility that GEIs are likely recruited via HGT to facilitate survival in an environment that is co-contaminated by heavy metals and aromatic compounds.

### 2.7. Genomic Comparison of Arthrobacter sp. Strain SRS-W-1-2016 

**Figure 7:**
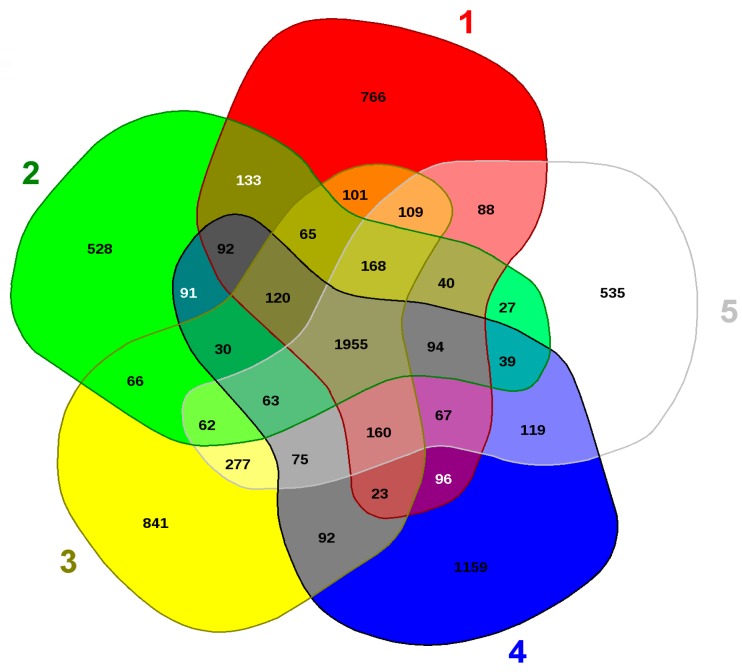
Shown are the whole genome-based Venn diagrams generated between *Arthrobacter* sp. SRS-W-1-2016 with its closest functional relatives. Venn diagram sectors belong to 1, *Phenarthrobacter aurescens* TC1; 2, *Arthrobacter cupressi* strain CGMCC; 3, *Arthrobacter globiformis* strain NBRC12137; 4, *Arthrobacter* sp. SRS-W-1-2016; and 5, *Pseudoarthrobacter phenanthrenivorans* strain Sphe3. The number of singleton genes appear in red, green, yellow, blue, and white areas for strains 1–5 listed above, respectively -along with their core genomes (centered gray area).

To further infer the evolutionary relatedness of strain SRS-W-1-2016 with close phylogenetic relatives, we ran the EDGAR analysis shown in Figure 7. This revealed that strain SRS-W-1-2016 harbors 1159 distinct genes that are not shared between the other four *Arthrobacter* species. Moreover, several distinctive genes, shown in parentheses, were also identified for each of the analyzed species: *Paenarthrobacter aurescens* TC1 (766); *A*. *cupressi* strain CGMCC1 (528); *A*. *globiformis* strain NBRC 1237 (841); and *Pseudoarthrobacter phenanthrenivorans* strain SWC37 (535), respectively. Notably, the SRS-W-1-2016—encoded distinct genes constituted approximately 26.7% of its total genome size, with much of this genome found to encode metal resistance proteins (e.g., for cadmium, cobalt, and zinc), transporter proteins, stress proteins, cytochromes, and drug resistance, respectively (Table 2). This comparative genomics analysis confirms the strong catabolic and bioremediation potential possessed by strain SRS-W-1-2016.

**Figure 8:**
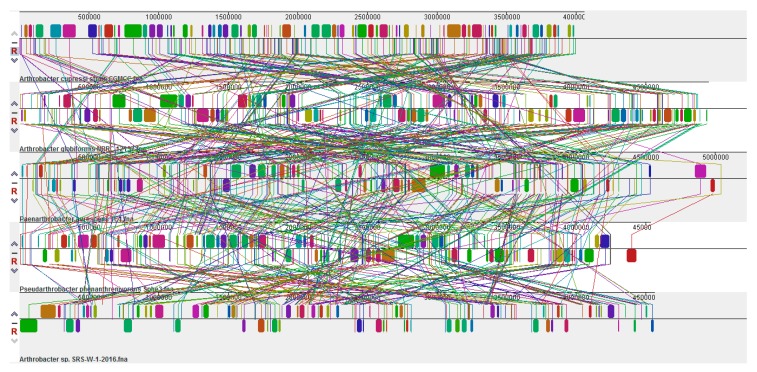
Whole genome comparative alignments between *Arthrobacter* sp. SRS-W-1-2016 with its closest functional relatives- *Pseudoarthrobacter phenanthrenivorans* strain Sphe3; *Phenarthrobacter aurescens* TC1; *A*. *globiformis* strain NBRC12137; and *A*. *cupressi* strain CGMCC. Each genome analyzed is presented horizontally with the scale showing the sequence coordinates and the conserved segments represented as the colored blocks which are connected across genomes. Blocks that are shifted downward in a genome represent those segments that are inverted relative to the reference genomes. The aligned region is in the forward orientation relative to the first genome sequence if a block lies above the center line; blocks below the center line indicate regions that align in the reverse complement (inverse) orientation of the reference genome. Genomic regions falling outside the blocks lack a detectable homology among the genomes analyzed.

Further comparative genomic analysis to identify the presence of large-scale evolutionary events, such as rearrangements and inversions, was performed by the multiple alignment of strain SRS-W-1-2016 with *Paenarthrobacter aurescens* TC1, *A*. *cupressi* strain CGMCC1, *A*. *globiformis* strain NBRC 1237, and *Pseudoarthrobacter phenanthrenivorans* strain SWC37, which revealed the presence of several crisscrossing locally collinear blocks (LCBs), suggesting the complicated rearrangement history of strain SRS-W-1-2016 relative to related *Arthrobacter* genomes. Regions that are inverted relative to strain SRS-W-1-2016 appear shifted below a genome’s center axis (Figure 8). In fact, visual inspection of the genomic rearrangement pattern for strain SRS-W-1-2016 relative to other *Arthrobacter* species revealed several instances of local overlapping inversions characteristic of symmetric inversion about the terminus, distinguishable as a “fan” pattern of crossing lines. Therefore, we hypothesize that the genomic collinearity of strain SRS-W-1-2016 was lost relative to its closest taxonomic relatives, most likely due to evolutionary mechanisms associated with genomic inversions and other rearrangements.

## 3. Experimental Section

### 3.1. Isolation and Physiological Growth Studies of Strain SRS-W-1-2016

Isolation of bacterial strains resistant to high concentrations of U and Ni, including *Arthrobacter* sp. SRS-W-1-2016, from uraniferous soil samples collected from the Tims Branch/ Steeds Pond area, was achieved following the methodology reported in our recent study [13]. Briefly, soils were serially diluted and 100 μL of this slurry was plated onto LB agar supplemented with U (4200 μM) and Ni (8500 μM), respectively. A rich growth media (LB) was used to isolate U and Ni resistant microorganisms because mineral salts media may have only selected a narrow range of soil microbiota and our intent was to obtain as many different groups of bacteria metal resistant bacteria as possible under the given experimental conditions. Because the strain was isolated on U and Ni concentrations that mimicked the soil concentrations, we evaluated growth on variable U and Ni concentrations using the Bioscreen C system (Growth Curves USA, Piscataway, NJ, USA). Briefly, 4M media [39] (modified with the addition of 0.04% yeast extract) was supplemented with U at an initial concentration of 100 μM that increased to a final concentration of 2000 μM, in increments of 100 μM, and Ni was tested at an initial concentration of 100 μM to a final concentration of 4000 μM, in increments of 200 μM, respectively. The assay was run using the honeycomb Bioscreen C plates containing 290 μL medium and 10 μL of the inoculum, which was grown overnight to an OD_600_ of 0.45 to 0.5. The instrument was programmed to perform regular shaking and capture OD_600_ at increments of every 4 h for four days; these experiments were run in triplicate and averaged values are reported. In addition to these studies, the whole genome sequence of strain SRS-1-W-2016 was used to evaluate in-silico phenotypic traits using Traitar (Microbial Trait Analyzer), which is a recently developed pipeline to predict 67 different traits of a bacterial genome sequence [34]; utilizing two different classification models (Phypat and Phypate + PGL) to present results in a single block.

### 3.2. Uranium Depletion by Strain SRS-W-1-2016

To determine U depletion by strain SRS-W-1-2016, microcosms were established in 4M media that was supplemented with 500 µM U and 1000 µM Ni, which was then inoculated with overnight grown culture to achieve a final OD_600_ of 0.05 ± 0.01. Flasks were incubated at 28 °C and 120 rpm for four days, with samples withdrawn every 24 h to determine the growth of bacterium (by OD_600_) and U depletion. Samples were acidified with HNO_3_ (1% *v*/*v*, final concentration) for determination of the spiked U and Ni, by inductively coupled plasma-mass spectrometry (ICP) on a NexIon 300 (Perkin Elmer, Inc., Waltham, MA, USA) in accordance with the quality assurance (QA) and quality control (QC) protocols of EPA method 6020B (USEPA, 2014; Method 6020B, Rev. 2. Inductively coupled plasma-mass spectrometry (ICP-MS). Office of Solid Waste, Washington, DC, USA).

### 3.3. Histochemical Screening of Strain SRS-W-1-2016 for Phosphatase Enzyme 

Histochemical plates amended with U were used for the detection of phosphatase activity during growth on U. Briefly, strain SRS-W-1-2016 was streaked onto LB agar plates (pH 7.0) containing TPMG [40]. Plates were incubated at 30 °C for 36 h. The bacterial phosphatase activity hydrolyses phenolphthalein diphosphate (PDP) present in the medium to phenolphthalein and phosphoric acid, which then results in intense bright green to bluish-green precipitates under acidic conditions due to the presence of methyl green (MG) dye (a pH indicator).

### 3.4. Nucleotide Sequence Accession Number

The draft genome sequence of *Arthrobacter* sp. strain SRS-W-1-2016 reported in this study has been deposited at DDBJ/ENA/GenBank under the accession MTPV00000000 (https://www.ncbi.nlm.nih.gov/nuccore/MTPV00000000); BioProject: PRJNA352261 (https://www.ncbi.nlm.nih.gov/bioproject/PRJNA352261); BioSample: SAMN06030390 (https://www.ncbi.nlm.nih.gov/biosample/SAMN06030390). The version described in this paper is version MSDV00000000.1.

### 3.5. Genomic Characterization of Strain SRS-W-1-2016 

Genomic DNA from strain SRS-W-1-2016 was extracted and prepared for sequencing on an Illumina HiSeq2000 instrument as described previously [13,41]. De novo assembly of the raw reads was performed with the SPAdes assembler [42] using default settings. Assembly coverage statistics were computed by mapping raw reads to the assembled genome using bowtie2 [43]. Specifically, we determined a coverage filter for the contigs from the distribution of coverage levels across the assembly. First, contigs were ordered by coverage, and cumulative assembly length was computed across all contigs. We then determined the coverage level at 50% of the total cumulative assembly length; half of that coverage level was selected as a coverage filter. The remaining reads were aligned with nucmer [42] against the complete genome sequence of *Arthrobacter* sp. Hiyo8 DNA (accession number AP014719.1), which was the closest taxonomic relative determined by a BLAST of the 16S rRNA sequence. All contigs aligned to these references, and the optimal contig ordering and orientation to most closely match the reference was determined using mummerplot [42] with the layout specified. Contigs were then reordered and reversed as needed to match the ordering determined by mummerplot.

A circular genomic map of strain SRS-1-W-2016 was generated using the CGView Comparison Tool [44]. Island Viewer was used to identify chromosomal deviations in GC content, known as genomic islands (GEIs) (http://www.pathogenomics.sfu.ca/islandviewer/) [35]. The genome, with a coverage of 90x, was then annotated and genes predicted by IMG/er [45], RAST [31], and National Center for Biotechnology Information’s (NCBI) Prokaryotic Genomes Automatic Annotation Pipeline (PGAAP), version 2.0. Resources utilized in this study for gene predictions and comparisons of strain SRS-1-W-2016 with other sequenced *Arthrobacter* spp., included those offered by NCBI [46] (http://www.ncbi.nlm.nih.gov/) and Integrated Microbial Genomes Expert Review [47] (https://img.jgi.doe.gov/cgi-bin/er/main.cgi). COGs from strain SRS-1-W-2016 and other *Arthrobacter* spp., were compared using the Function Category Comparison tool using IMG/ER. IMG computes top COG hits using RPS-BLAST on PSSM’s provided by CDD. A genome-based phylogenetic tree of strain SRS-1-W-2016 was constructed using the One Codex database platform [48], which generates taxonomic classification of nucleotide reads by assessing for exact k-mer matches against their database of bacterial, viral, and fungal genomes, as well as the NCBI’s Reference Sequence Database.

### 3.6. Characterization of the Resistome of Strain SRS-W-1-2016 

Antibiotic resistance genes (the resistome), present in strain SRS-1-W-2016, were identified using the Comprehensive Antibiotic Resistance Database (“CARD”) pipeline [49]. CARD is a repository of curated sequences and SNPs assembled according to the Antibiotic Resistance Ontology (ARO). Biosynthetic gene clusters (BCs) represented within the genome of strain SRS-1-W-2016 were identified using the IMG-ABC (https://img.jgi.doe.gov/abc) pipeline, which is a repository of previously characterized BCs and associated secondary metabolites (SMs), respectively [50]. Protein families (pfams) within each of the BC were downloaded after IMG-ABC analysis and complete linkage hierarchical clustering was performed in R (https://www.r-project.org/) using a Euclidean distance metric; clustered results were visualized in a heatmap. Additionally, the recently updated “antibiotics and secondary metabolite analysis shell”—antiSMASH pipeline was also utilized to mine the genome of strain SRS-1-W-2016 for the presence of both the resistome and metabolite gene clusters [51].

### 3.7. Comparative Genomics of Strain SRS-W-1-2016

Initial comparative genomics of strain SRS-1-W-2016 with closest taxonomic relatives was performed by EDGAR [32]. To further infer the evolutionary relatedness of SRS-1-W-2016 with its closest taxonomic relatives, multiple alignments were performed using the progressive Mauve algorithm (http://darlinglab.org/mauve/mauve.html) [52]. Mauve facilitates multiple genome alignments such that rearrangements and inversions from evolutionary events can be identified and comparatively visualized. Because genomic recombination events result in rearrangements, orthologous genomic regions of a bacterial strain may be reordered or inverted relative to another genome, which are clearly identified during Mauve analysis such that conserved genomic segments that appear to be internally free from rearrangements are shown as Locally Collinear Blocks (LCBs).

Average nucleotide identity was obtained as shown previously [53] (http://enve-omics.ce.gatech.edu/ani/) and dDDH was estimated by using the Genome-to-Genome Distance web service [54] (http://ggdc.dsmz.de/home.php), respectively. The ANI calculated utilized both best hits (one-way ANI) and reciprocal best hits (two-way ANI) between two genomic datasets, as shown by Goris et al. [55]. Typically, the ANI values between genomes of the same species are above 95%. GGDC runs comparisons of a query genome relative to a reference genome and generates an intergenomic distance under three different distance formulae. Distances are inferred using three distinct formulas from the set of high-scoring segment pairs (HSPs) and maximally unique matches (MUMs) obtained by comparing each pair of genomes with the chosen software. These distances are transformed to values analogous to DDH using a generalized linear model (GLM) inferred from an empirical reference dataset comprising real DDH values and genome sequences. Model-based confidence intervals are specified in square brackets but can also be obtained via bootstrapping. Logistic regression (a special type of GLM) is used for reporting the probabilities that DDH is ≥70% and ≥79%. Percent G+C content cannot differ by >1 within a single species but by ≤1 between distinct species.

## 4. Discussion

Overall, this study advances our understanding on the genome-wide mechanisms potentially employed by the soil-borne *Arthrobacter* strain SRS-W-1-2016 to resist and survive in a highly contaminated habitat. Moreover, environmental microorganisms underpin metal biotransformations, including radionuclide precipitation, sorption, intracellular accumulation, and biomineralization, and therefore, such studies enable a better understanding of genes that facilitate bacteria to not only successfully colonize such harsh environments, but also provides a sustainable approach to remediate toxic habitats. By engaging in horizontal gene transfer mechanisms, environmental microorganisms recruit ecologically relevant traits such as metal binding proteins, permeases, transport regulators, and efflux pumps that can mediate the shunting of metals to the extra-cellular environment or prevent their cellular uptake, so the bacterium can flourish under the stress of toxic contaminants. Furthermore, it also appears that metal stress can inadvertently enhance the bacterium’s resistome, i.e., the arsenal of antibiotic resistance genes, as demonstrated in a long-term Ni contamination study [56]. This heavy metal-driven co-selection has the propensity to further intensify the evolutionary processes, leading to the development of highly potent multi-drug resistant ‘superbugs’ with genomic attributes that cause human and animal diseases. Overall, this study forms the basis for obtaining a comprehensive understanding on the genomic mechanisms of metals bioremediation and/or resistance and thereby can potentially provide better management and stewardship of historically polluted habitats, such as the U.S. DOE managed SRS ecosystem.

## Figures and Tables

**Table 1:** Functional traits connected with Kyoto Encyclopedia of Genes and Genomes (KEGG) pathways identified from the whole genome sequence of *Arthrobacter* sp. strain SRS-W-1-2016. This figure was prepared by analysis of the whole genome using the integrated microbial genome expert review (IMG/er) and values were plotted using Microsoft Excel.

KEGG Category	Percentage (%)
Global and Overview Maps	63.08
Carbohydrate Metabolism	25.27
Amino Acid Metabolism	22.78
Energy Metabolism	12.01
Metabolism of Cofactors and Vitamins	11.39
Membrane Transport	9.34
Nucleotide Metabolism	8.72
Lipid Metabolism	7.56
Translation	7.21
Xenobiotics Biodegradation and Metabolism	6.32
Signal Transduction	5.87
Metabolism of other Amino Acids	4.8
Replication and Repair	4.54
Folding, Sorting and Degradation	4.18
Metabolism of Terpenoids and Polyketides	3.91
Biosynthesis of other Secondary Metabolites	3.2
Glycan Biosynthesis and Metabolism	2.85
Cell Motility	2.67
Cell Growth and Death	1.42
Drug Resistance	1.33
Transport and Catabolism	0.98
Transcription	0.44
Environmental Adaptation	0.18
Excretory System	0.18

**Table 2:** Shown are PROKKA-annotated gene homologues that likely perform a biodegradative or metal resistance function in *Arthrobacter* sp. strain SRS-W-1-2016. The analysis was conducted by manually querying the set of 1159 genes that were identified only in *Arthrobacter* sp. strain SRS-W-1-2016, for desired bioremediation functions.

Category	Gene Homologue
Transporter proteins	Low-affinity inorganic phosphate transporter
	H(+)/Cl(-) exchange transporter *ClcA*
	Putative inner membrane transporter *YhbE*
	Divalent metal cation transporter *MntH*
Stress proteins	General stress protein 14
	Universal stress protein/MT2085
Cytochromes	Cytochrome P450 107B
	Cytochrome bc complex cytochrome b subunit
Metal resistance proteins	Cadmim, cobalt and zinc/H(+)-K(+) antiporter
	Arsenical resistance operon repressor
	Copper resistance protein A precursor
	Copper chaperone *CopZ*
Drug resistance	Multidrug resistance operon repressor
	Multidrug resistance protein

## Supplementary Materials

The following are available online at www.mdpi.com/2073-4425/9/1/31/s1.
